# Sleep deprivation reduces the baroreflex sensitivity through elevated angiotensin (Ang) II subtype 1 receptor expression in the nucleus tractus solitarii

**DOI:** 10.3389/fnins.2024.1401530

**Published:** 2024-04-29

**Authors:** Ling-feng Liu, Yu-wan Wang, Jia-cen Sun, Yang-kai Wang, Xing Tan, Wei-zhong Wang

**Affiliations:** ^1^School of Medicine, Shanghai University, Shanghai, China; ^2^Department of Marine Biomedicine and Polar Medicine, Naval Medical Center, Naval Medical University (Second Military Medical University), Shanghai, China; ^3^Key Laboratory of Medical Electrophysiology of Ministry of Education, Medical Electrophysiology Key Lab of Sichuan Province, Institute of Cardiovascular Research, Southwest Medical University, Luzhou, China

**Keywords:** baroreflex sensitivity, AT1R, NTS, sleep deprivation, cardiovascular diseases

## Abstract

**Introduction:**

Sleep insufficiency has been linked to an increased risk of high blood pressure and cardiovascular diseases. Emerging studies have demonstrated that impaired baroreflex sensitivity (BRS) is involved in the adverse cardiovascular effects caused by sleep deprivation, however, the underlying mechanisms remain unknown. Therefore, the present study aims to clarify the role of abnormal renin-angiotensin system in the nucleus tractus solitarii (NTS) in impaired BRS induced by sleep deprivation.

**Methods:**

Rats were randomly divided into two groups: normal sleep (Ctrl) and chronic sleep deprivation (CSD) group. Rats were sleep deprived by an automated sleep deprivation system. The blood pressure, heart rate, BRS, the number of c-Fos positive cells and the expression of angiotensin (Ang) II subtype 1 receptors (AT1R) in the NTS of rats were assessed.

**Results:**

Compared to Ctrl group, CSD group exhibited a higher blood pressure, heart rate, and reduced BRS. Moreover, the number of c-Fos positive cells and local field potential in the NTS in CSD group were increased compared with the Ctrl group. It was shown that the expression of the AT1R and the content of Ang II and the ratio of Ang II to Ang-(1–7) were increased in the NTS of rats in CSD group compared to Ctrl group. In addition, microinjection of losartan into the NTS significantly improved the impaired BRS caused by sleep deprivation.

**Discussion:**

In conclusion, these data suggest that the elevated AT1R expression in the NTS mediates the reduced BRS induced by chronic sleep deprivation.

## Background

Maintaining normal sleep is essential for human health, and people spend about one-third of their day sleeping. It is increasingly recognized as an important lifestyle behavior that can affect cardiovascular disease and mortality in populations (Blodgett et al., 2023; Lane et al., 2023). In addition, sleep insufficiency has become a prevalent health problem in developed countries (Wang et al., 2019). Sleep deprivation has profound effects on many aspects of physiological functioning, such as alertness, cognition, mood, immune function, and autonomic nervous activity (Dettoni et al., 2012; DelRosso et al., 2020). High cardiovascular incidence has been repeatedly reported in shift workers, whose sleep is often disturbed and insufficient (Kanki et al., 2023). Moreover, inadequate sleep has been linked to an increased risk of high blood pressure and cardiovascular diseases (Fan et al., 2020). These phenomena have been reported to be mediated primarily by reducing energy expenditure, upregulating appetite, and altering glucose metabolism (Liu et al., 2022). However, the exact mechanism of the high incidence of cardiovascular disease due to insufficient sleep has not been well elucidated. The evidence from these studies shows inconsistencies in the relationship between sleep and cardiovascular disease, and there is not enough impetus for mechanistic analysis. Therefore, the study aims to focus on elucidating the mechanism by which sleep deprivation causes cardiovascular effects.

Arterial blood pressure generally drops during sleep, usually between 20 and 30 mmHg. Moreover, studies have reported that sleep is also associated with increased baroreflex sensitivity (BRS) (Legramante et al., 2003; Kaiser et al., 2022). Baroreflex, a basic neural feedback mechanism that regulates cardiovascular function, is modulated by sleep–wake transitions (Conway et al., 1983). The nucleus tractus solitarii (NTS), an integrative nucleus that communicate with main centers, is involved in autonomic control that participate in the regulation of sympathetic outflow and blood pressure (Guyenet, 2006). However, it is unclear whether the neural mechanisms involved in BRS in the NTS can be regulated by sleep deprivation.

The role of the brain renin-angiotensin system (RAS), particularly the angiotensin (Ang) II subtype 1 receptors (AT1R), in the regulation of blood pressure and sympathetic tone has been well established (Souza and Earley, 2022). Brain Ang II, acting on AT1R in different parts of the brain, including the paraventricular nucleus (PVN) of the hypothalamus and rostral ventrolateral medulla (RVLM), increases blood pressure and sympathetic nerve activity (Deng et al., 2019). There is clear evidence that brain Ang II has a sympathoexcitatory effect through the stimulation of AT1R on spinally projecting glutamatergic neurons located in the RVLM. Reportedly, microinjection of Ang II into the NTS reduces BRS (Casto and Phillips, 1986). The endogenous Ang II exerts a tonic inhibitory modulation on BRS response via an action on AT1R but not subtype 2 receptors (AT2R) in the NTS (Luoh and Chan, 1998). More interestingly, numerous studies have shown that RAS is significantly activated in sleep-deprived animals and in patients with sleep apnea (Visniauskas et al., 2011; Loh et al., 2023). It is suggested that abnormal Ang II is also a significant pathological mediator in the brain after sleep disorders, but whether it is involved in BRS injury is unclear.

Taken together, the present study aimed to explore the potential mechanisms of the cardiovascular effects of sleep deprivation. We have reported our findings that elevated AT1R expression in the NTS is associated with cardiovascular effects of sleep deprivation. These findings will provide more insight into the effects of sleep deprivation on cardiovascular diseases.

## Methods

### Animals

A total of 70 male Sprague–Dawley rats (8–12 weeks old, 200–220 g) were used in this study. The rats were purchased from the Experimental Animal Center of Naval Medical University and housed in a temperature and humidity controlled, 12–12 h light–dark cycle animal room. All experimental protocols followed the ethical guidelines and received approval from the Animal Use and Care Committee of the Naval Medical University (IACUC protocol number: NMU-20210901).

### Chronic sleep deprivation procedure

The protocol of chronic sleep deprivation (CSD) was described according to previous studies (Wang et al., 2022; Yang et al., 2022). Rats were sleep deprived by an automated sleep deprivation system (Shanghai XinRuan Information Technology Co., Ltd. Shanghai, China) with a motorized rotating bar on the bottom of the cage. Before starting the CSD procedure, rats were acclimated to the automated sleep deprivation system for 7 days to minimize stress. The automated sleep deprivation system reduced the sleep of rats by randomly rotating bars without significantly increasing their movement. In brief, rats were housed in cages with a rotating bar which was turned on at 15:00 p.m. and turned off at 11:00 a.m. am the next day, and kept continuously for 20 h. The entire CSD program was performed continuously for 21 days. After sleep deprivation, rats were permitted to sleep at 11:00 a.m. and maintain for 4 h every day. While the control rats stay in the same system, they were allowed to sleep normally at the corresponding time.

### Measurements of blood pressure and BRS

As described in our previous study (Tan et al., 2022), rats were anesthetized with urethane (800 mg/kg, ip) and α-chloralose (40 mg/kg, ip). The mean arterial pressure (MAP)/heart rate (HR) were recorded in anesthetized rats by femoral artery cannulation using Power Lab system (AD Instruments, Australia). The method for measuring BRS was based on previous studies reported (Lu et al., 2015; Jun et al., 2023). Briefly, rats were anesthetized with urethane (800 mg/kg, ip) and α-chlorose (40 mg/kg, ip). Bradycardia was induced by intravenous injection of the α-adrenergic receptor agonist phenylephrine hydrochloride (PE; Sigma, United States). PE (25 μg/kg) injected intravenously to elevate arterial blood pressure and induces bradycardia. For each response, the change in heart rate (∆HR) to the change in blood pressure (∆MAP) was used as an index of BRS.

### PE induced c-Fos expression

To minimize c-Fos expression triggered by prolonged wakefulness, we performed surgery in CSD rats after 4 h of sleep and used continuous stimulation to induce c-Fos expression in baroreflex sensitive neurons. Briefly, femoral vein catheter was connected to a syringe pump, which was filled with PE dissolved in 0.9% sterile saline at a dose of 0.5 mg/mL. Infusion was performed at 17–21 μL/min for 60 min to raise MAP by approximately 40 mmHg. The low infusion rate was used to minimize mechanical stimulation of cardiac volume receptors as well as fluid loading, whereas the duration was chosen to provide a stimulus prominent enough to induce c-Fos expression (Chaudhary et al., 2021). The immunofluorescence staining was performed as described previously (Wu et al., 2016). Sections were washed three times for 10 min in PBS and then incubated with 5% bovine serum albumin containing 0.3% Triton X-100 at room temperature for 2 h. after that the sections were incubated overnight with rabbit monoclonal anti-c-Fos antibody (No. ab214672, Abcam). The next day, sections were washed by PBS and incubated with goat anti-rabbit IgG conjugate with AlexaFluor594 (1:200, Jackson ImmunoResearch, United States) for 2 h at room temperature. Sections were photographed under a fluorescence microscope (Leica, Germany) and counted by ImageJ. In brief, outlines of the brain regions to be counted were drawn using Adobe Illustrator software according to the brain atlas (Paxinos and Watson, 1998). All images were overlaid with the corresponding atlas section to anatomically define the regions of interest. All c-Fos labeled cells within the boundaries of the defined sites were marked using ImageJ software. Brightness and contrast were optimized for each image. Background subtraction was performed by subtracting the mean intensity value estimated from a single background regions of interest placed within an unlabeled region in the same image. The c-Fos signals were analyzed as separate images taken from the same slice using their excitation wavelengths. In order to be counted positive, a cell had to display an intensity value above the intensity threshold of the background.

### *In vivo* electrophysiology

Rats were anesthetized using isoflurane (oxygen flow meter: 0.8–1.5 L/min, isoflurane vaporizer: 1.5–3%). The rat was mounted in the stereotaxic device (RWD Life Science, China). The skin of the head surface was swabbed using alcohol and iodine, and made a cut. The fascia was gently scraped from the top of the skull with a sterile cotton swab. Six stainless steel screws were implanted into the top of the skull. According to the atlas of Paxinos and Watson (1998), a small hole was drilled 0.5 mm lateral and 15.3 mm posterior to the bregma. The underlying dura mater was punctured and moved aside. The multiple-electrode arrays (Teflon-coated stainless-steel wires, 25 mm diameter, 8 channels, Kodou, China) was fixed to the stereotactic device with clips, and then the electrode bundle was slowly implanted 5.5 mm below the surface of the cerebellum, and after that the electrodes were fixed to the head with dental cement. After the recovery from surgery, the headstage was connected to the electrodes in the rat’s head for signal acquisition and analysis. Briefly, electrodes were connected to a Plexon multichannel acquisition system (OmniPlex-D, PLEXON, Hong Kong) to record local field potential (LFP). Recordings were started at 24 h post-surgery to allow the animals to recover. Before data collection rats were injected PE (2 mg/kg) intraperitoneally. The LFP signals were first low-pass filtered with a second-order Butterworth filter from 0.5 to 200 Hz, and then sub-sampled with a Plexon multichannel acquisition system at 1 kHz. Matlab R2023a (Mathworks) was used to analyze the LFP data. To minimize edge artifacts, 10 s of data were removed from the beginning of each trial. Power spectral density (PSD) was analyzed for 200 s per sample using Welch’s formula. The power of *δ* (1–4 Hz), θ (4–8 Hz), *α* (8–15 Hz), *β* (15–30 Hz), low *γ* (30–60 Hz), and high *γ* (60–100 Hz) rhythms was analyzed in the electroencephalogram (EEG). These data were calculated by averaging each channel of the animal (Liu et al., 2021).

### Total RNA extraction and quantitative polymerase chain reaction

According to our previous study (Wu et al., 2016), the tissues of NTS were collected by punching. The tissue from a single rat was recorded as one sample. Total RNA of NTS tissue was extracted with TransZol UP reagent (TransGen Biotech, China), and then RNA was reverse-transcribed to cDNA using a reverse transcription kit (TransGen Biotech, China). The cDNA was amplified by SYBR Green qPCR Mix (TransGen Biotech, China). Quantitative PCR amplification was performed on LightCycler96 Instrument (Roche, United States) using the two-step PCR amplification standard program. The relative expression was calculated by the 2^−ΔΔCt^ method and normalized to GAPDH. The primer sequences used in present study are shown below. GAPDH: Forward primer GACATGCCGCCTGGAGAAAC, Reverse primer AGCCCAGGATGCCCTTTAGT; AT1R: Forward primer GCCAAAGTCACCTGCATCAT, Reverse primer AATTTTTTCCCCAGAAAGCC.

### Western blotting

Based on our previous study (Duan et al., 2022), NTS tissues were punched as protocol in quantitative polymerase chain reaction (qPCR). The tissue of NTS was lysed with lysate (Beyotime, China). The protein concentration of NTS sample was measured by BCA Protein Assay Kit (Beyotime, China), diluted with 5× SDS-PAGE uploading buffer (Solarbio, China) and PBS (Solarbio, China) to denature by boiling. Proteins were separated by SDS-PAGE in the 10% gradient gel (EpiZyme, China) and transferred to a PVDF membrane (Sigma, United States). After transfer, the PVDF membrane was incubated with 5% skimmed milk (EpiZyme, China) in TBST for 1.5 h at room temperature. The primary antibody (anti-AT1R, No. ab124734, Abcam) was diluted at 1:1000 in TBST, and then the PVDF membrane was incubated overnight at 4°C. The PVDF membrane was washed 3 times and then incubated with the secondary antibody for 2 h at room temperature. And the immunostaining bands were detected by an automatic chemiluminescence image analysis system (Tanon Science & Technology, China).

### Enzyme-linked immunosorbent assay

As described above, performing protein extraction and quantification of NTS tissue. The concentrations of Ang II and Ang-(1–7) in the NTS were quantified using an enzyme-linked immunosorbent assay (ELISA) kit (AiFang biological, China). The method was done according to the manufacturer’s instructions.

### NTS microinjection

The procedure of losartan microinjection in the NTS was based on our previous study (Wang et al., 2007). In brief, the anaesthetized rat was fixed in a stereotaxic frame. And the dorsal surface of the medulla oblongata was exposed via resecting cervical muscles and occipital bone. Microinjection was performed using the micropipette using a pressure syringe (World Precision Instruments, United States) with a pipette tip diameter of approximately 20–30 μm. Functional localization was performed by a rapid depressor effect after injecting L-glutamate (2 nmol, 50 nL) to determine the dorsomedial NTS coordinates relative to the calamus scriptorius: rostral 0.4–0.5 mm, lateral 0.5–0.6 mm, and deep 0.4–0.5 mm. Losartan (100 pmol, 50 nL), an AT1R blocker, and L-glutamate was dissolved in artificial cerebrospinal fluid (aCSF). The effects of bilateral microinjection of losartan into the NTS on BRS were observed. About 30 min after the first BRS test, the rats’ blood pressure and HR returned to the level before the BRS measurement. Next, bilateral microinjections of losartan into the NTS were performed. The aCSF was used as a solvent control. Finally, in order to observe the effect of microinjection of losartan into the NTS on BRS, the BRS tests were performed again at 10 min after microinjection of losartan into NTS.

### Statistical analysis

Data are expressed as mean ± standard error of the mean (SEM). Comparisons between the control and experimental groups were made using the student’s *t*-test (paired and unpaired). The statistical significance of the differences was considered when *p*-values were less than 0.05. All statistical analysis was carried out using 9.1 Version GraphPad Prism software.

## Results

### The effects of sleep deprivation on cardiovascular functions

**Figure 1:**
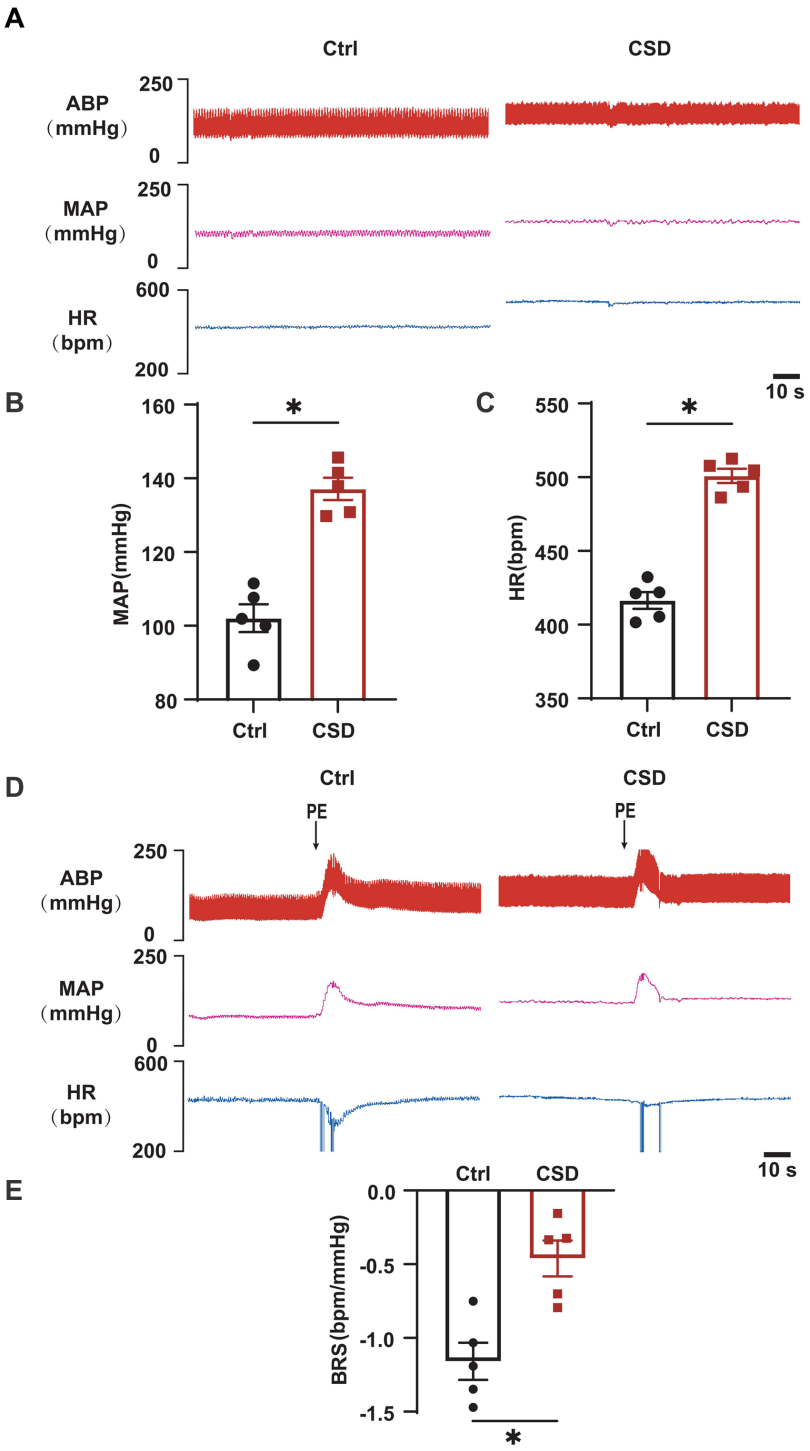
Effects of chronic sleep deprivation on MAP, HR, and BRS. **(A)** Representative tracings of blood pressure and HR in control and chronic sleep deprived rats. **(B,C)** Statistical graphs of MAP **(B)**, HR **(C)**. **(D)** Representative tracings of BRS in the control and chronic sleep deprived rats. **(E)** Statistical graph of BRS in the control and chronic sleep deprived rats. ^*^p < 0.05 vs. Ctrl group; *n* = 5/group.

To observe the effects of sleep deprivation on cardiovascular function in rats, MAP and HR were recorded, and the BRS was assessed by intravenous PE. As shown in Figure 1, compared to Ctrl group, CSD group exhibited a higher blood pressure (MAP, 102.1 ± 3.8 mmHg vs. 133.7 ± 2.8 mmHg, *p* < 0.05), HR (416.5 ± 5.7 bpm vs. 500.9 ± 4.8 bpm, *p* < 0.05), and reduced BRS (−1.16 ± 0.13 bpm/mmHg vs. −0.46 ± 0.12 bpm/mmHg, *p* < 0.05).

### The effects of sleep deprivation on the activity of neurons in the NTS

**Figure 2:**
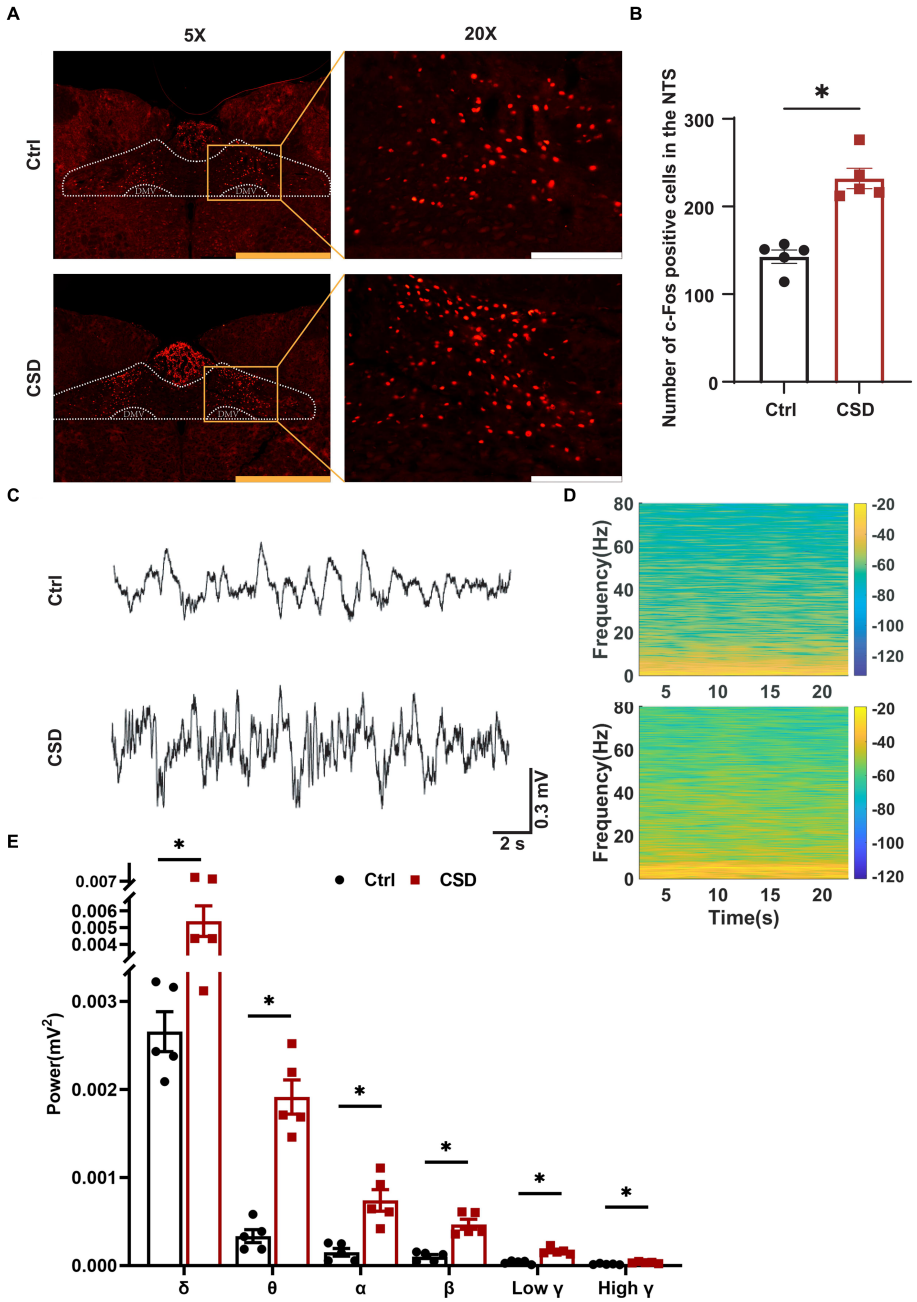
Effect of chronic sleep deprivation on the number of PE-induced c-Fos expression and LFP in the NTS. **(A)** Representative images of PE-induced c-Fos positive cells in control and chronic sleep deprived rats in the NTS. The yellow scale bar was 800 μm, the white scale bar was 200 μm. **(B)** Statistical graph of PE-induced c-Fos positive cells in control and chronic sleep deprived rats in the NTS. **(C)** Representative traces of electroencephalography recording in the NTS of control and chronic sleep deprived rats. **(D)** Representative spectrogram in the NTS of control and chronic sleep deprived rats. **(E)** Statistical data for power among *δ*, *θ*, *α*, *β*, low *γ*, and high *γ*. ^*^p < 0.05 vs. Ctrl group; *n* = 5/group.

**Figure 3:**
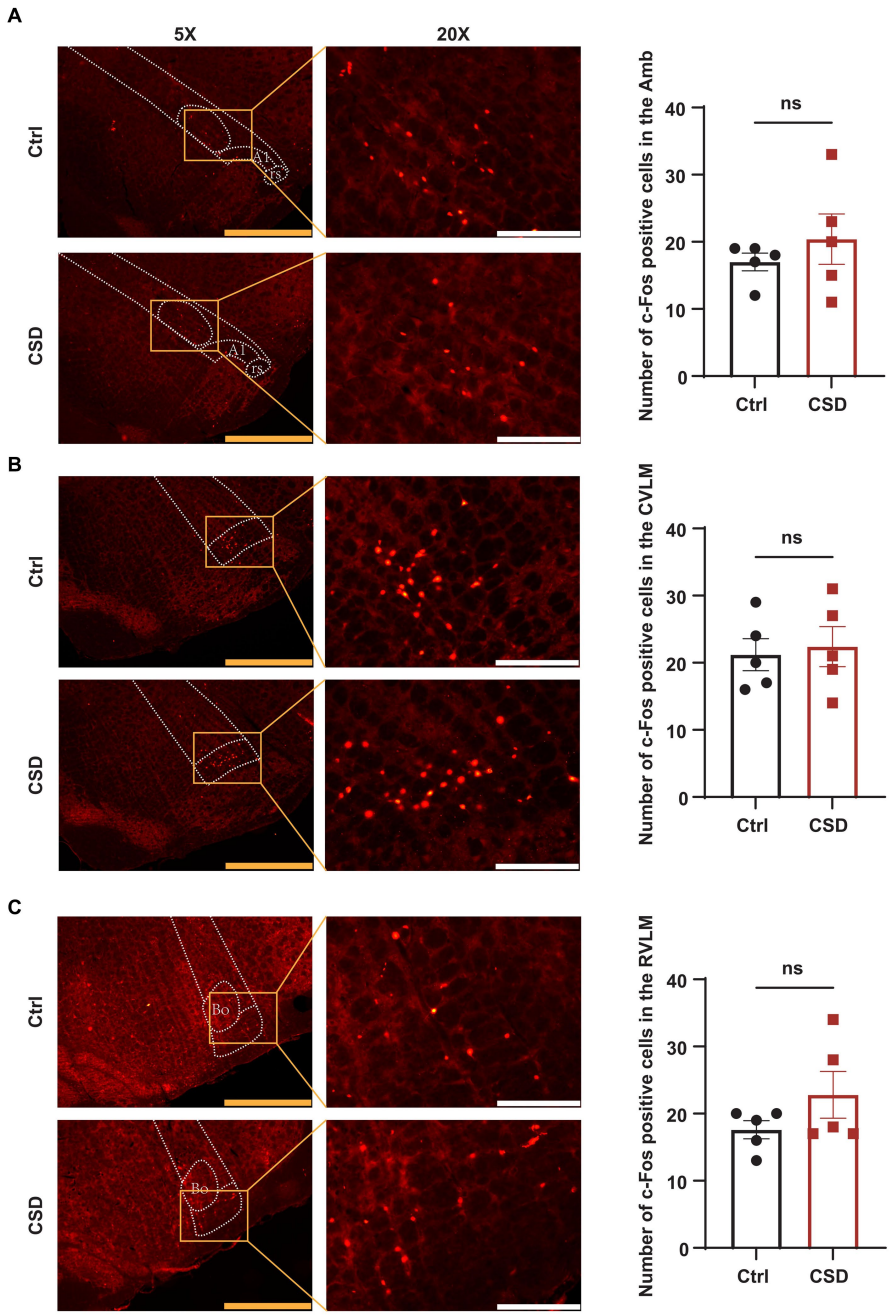
Effect of chronic sleep deprivation on the number of PE-induced c-Fos expression in the brain regions associated with the regulation of autonomic nervous function. **(A–C)** Representative images of PE-induced c-Fos positive cells in control and chronic sleep deprived rats in the Amb (−14.52 to −14.40 relative to Bregma), CVLM (−14.16 to −14.04 relative to Bregma), and RVLM (−12.24 to −12.12 relative to Bregma) with respective statistical graphs. The yellow scale bar was 800 μm, the white scale bar was 200 μm, *n* = 5/group.

To observe the effects of sleep deprivation on the activity of neurons in the NTS of rats, we labeled c-Fos positive neurons and recorded the field potential in the NTS. It was found that the number of c-Fos positive cells (Figures 2A,B) and the LFP (Figures 2C–E) induced by PE were increased significantly in the NTS of rats in CSD group. However, compared with control rats, the numbers of c-Fos positive neurons in the nucleus ambiguus (Amb), caudal ventrolateral medulla (CVLM), and RVLM of rats with sleep deprivation were not changed significantly (Figure 3).

### The effects of sleep deprivation on the RAS in the NTS

**Figure 4:**
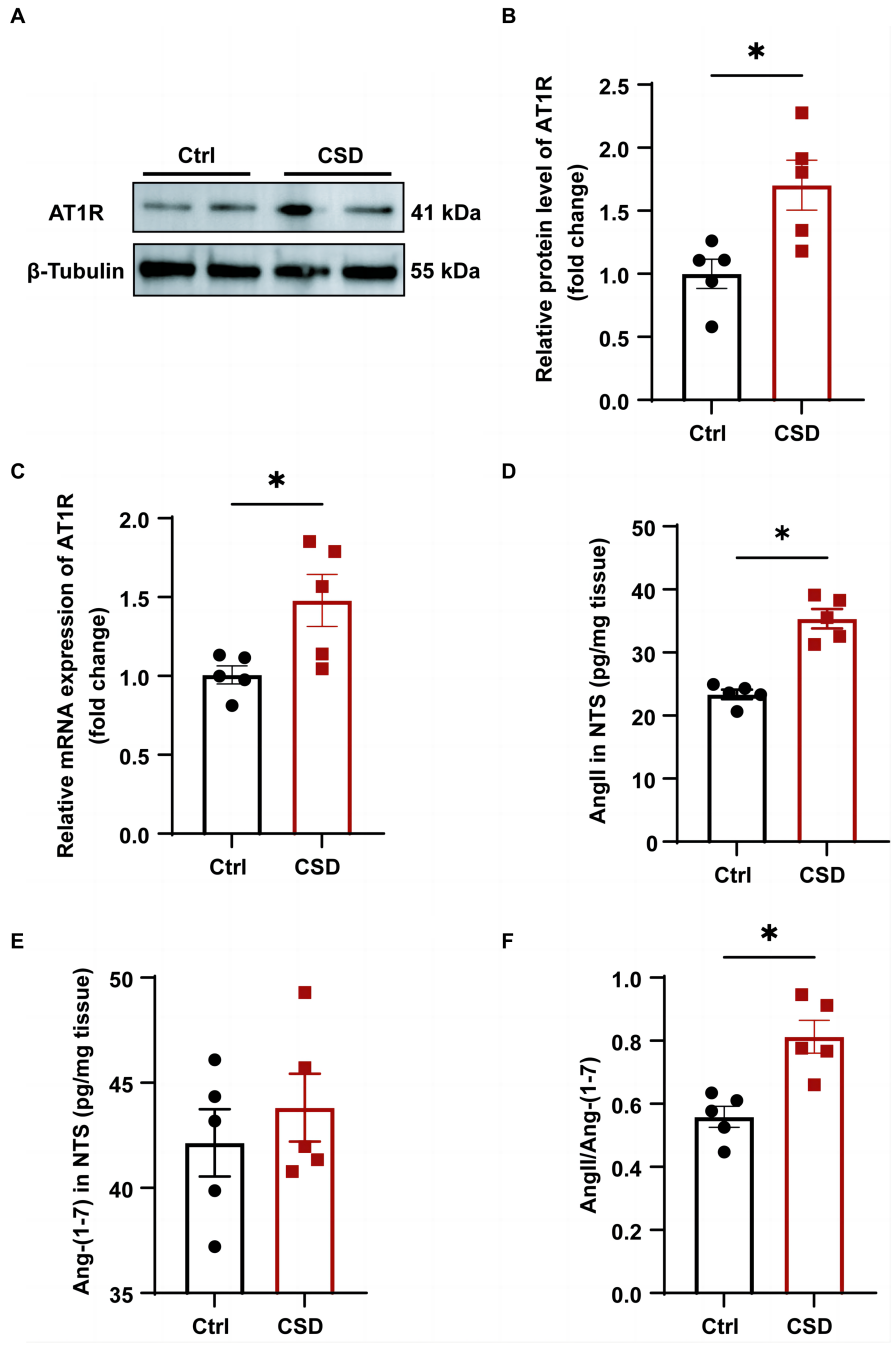
The effects of chronic sleep deprivation on the RAS in the NTS. **(A)** The protein levels of AT1R in the NTS was determined by western blotting from different parts of the same gel. **(B)** Bar graph representing the protein expression of AT1R in the NTS. **(C)** The expression of AT1R in the NTS of control and chronic sleep deprived rats was determined by qPCR. **(D–F)**. The concentration of Ang II **(D)** and Ang-(1–7) **(E)** and the ratio of Ang II to Ang-(1–7) **(F)** in NTS tissues of two groups. ^*^p < 0.05 vs. Ctrl group; *n* = 5/group.

To further explore the molecular mechanisms involved in sleep deprivation leading to abnormal cardiovascular function, the changes in the components of RAS in the NTS were evaluated. As shown in Figure 4, the protein (Figures 4A,B) and mRNA (Figure 4C) expression of the AT1R were increased significantly in the NTS of rats in CSD group compared with the Ctrl group. Although the level of Ang-(1–7) (42.14 ± 1.598 pg/mg vs. 43.82 ± 1.618 pg/mg, *p* > 0.05) in the NTS of rats was not changed between the two groups, and the level of Ang II (23.36 ± 0.74 pg/mg vs. 35.35 ± 1.54 pg/mg, *p* < 0.05) and the ratio of Ang II to Ang-(1–7) (0.56 ± 0.03 vs. 0.81 ± 0.05, *p* < 0.05) were increased significantly after sleep deprivation (Figures 4D–F).

### The effects of losartan on impaired BRS in CSD

**Figure 5:**
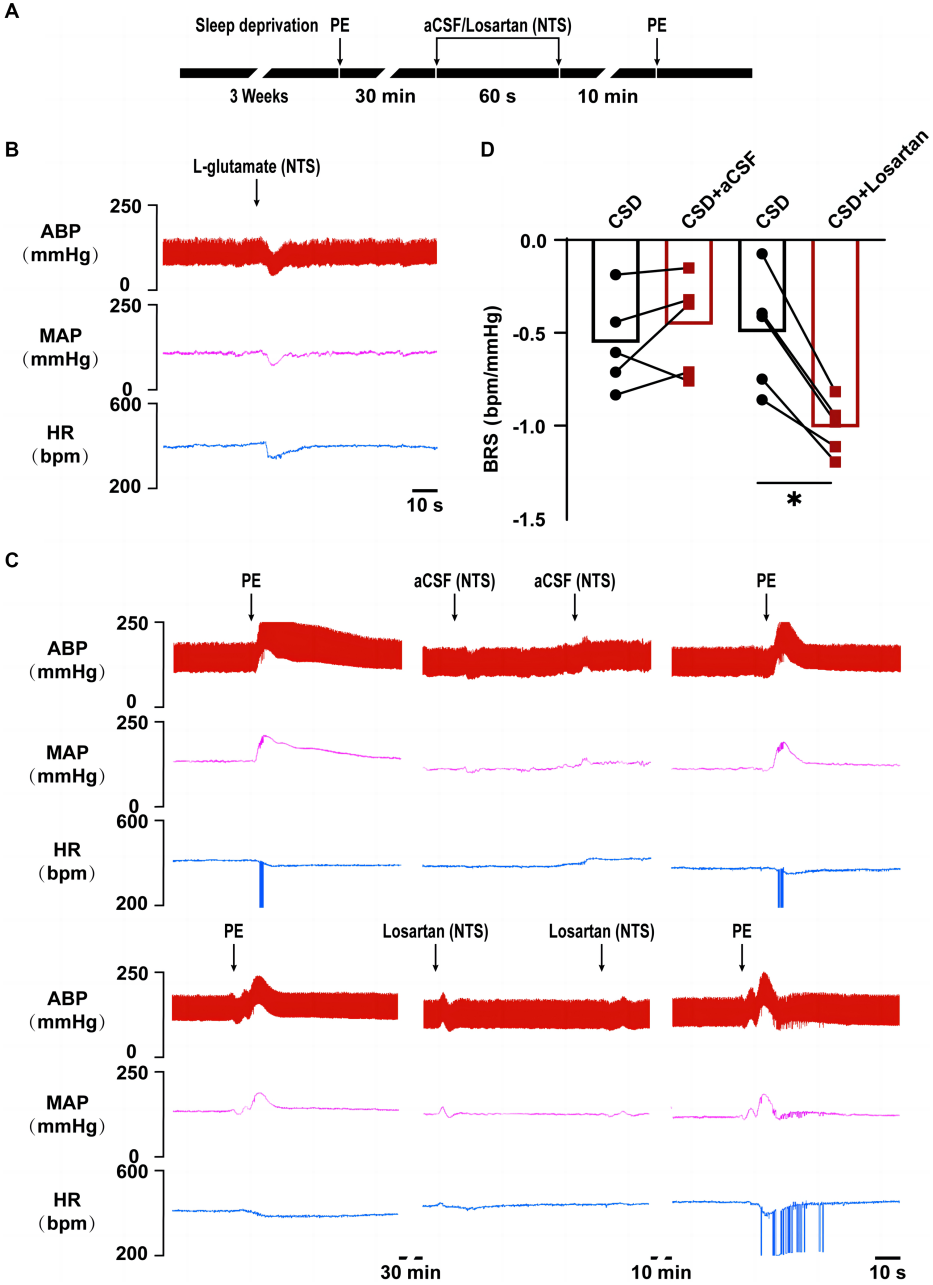
The effect of NTS microinjection of losartan on BRS in chronic sleep deprived rats. **(A)** Experimental design for microinjection of aCSF or losartan into the NTS of rats in chronic sleep deprivation. **(B)** Representative tracings of NTS functionally identification. **(C)** Representative tracings of BRS before and after microinjections of aCSF and losartan into the NTS of chronic sleep deprived rats. **(D)** Statistical graph of BRS in two groups before and after microinjections of aCSF or losartan. ^*^p < 0.05 vs. Ctrl group; *n* = 5/group.

*In vivo* experiment, compared with the changes in BRS (−0.56 ± 0.11 bpm/mmHg vs. −0.50 ± 0.14 bpm/mmHg, *p* > 0.05) after microinjection of aCSF into the NTS, microinjection of losartan increased BRS in CSD (−0.46 ± 0.12 bpm/mmHg vs. −1.01 ± 0.07 bpm/mmHg, *p* < 0.05) (Figure 5).

## Discussion

It is well known that the transition from wakefulness to sleep is associated with profound changes in both the brain state and the motor activity of the animal. Additionally, there is a decrease in somatic activity and skeletal muscle tone, as well as a decrease in autonomic activity, resulting in lower blood pressure and slower heart rate (Chouchou et al., 2013). Cardiovascular diseases such as hypertension and coronary artery disease are significantly worsened by insufficient sleep (Vishnu et al., 2011). However, the mechanistic link between sleep deprivation and cardiovascular health remains poorly understood, especially at the neural circuit level. In our study, sleep-deprived rats had increased blood pressure and heart rates while their BRS decreased. At the same time, with the increase of the activity of neurons, the field potential was enhanced and the expression of AT1R in the NTS was increased. Further blockade of AT1R in the NTS significantly attenuated the reduction of BRS induced by sleep deprivation. These results suggest elevated AT1R expression in the NTS mediates the impaired BRS induced by sleep deprivation. Through the lens of neural regulation, these findings will shed more light on the prevention and treatment of cardiovascular diseases and the risk factors related to sleep deprivation.

Several studies demonstrated that both acute and chronic sleep loss may affect the cardiovascular system, the immune responses, the hormonal pathways and thermoregulation (Tenorio et al., 2013; Kanki et al., 2023). Subjects exposed to sleep restriction showed an increased activation of the stress response systems, such as the autonomic nervous system, the hypothalamic–pituitary–adrenal axis and the immune system (Meerlo et al., 2008). Based on evaluation of the cardiovascular effects of sleep deprivation, a novel finding in the present study is that in addition to pressor response, chronic sleep deprivation could also cause BRS impairment. This result suggests that there is a problem in a certain node of the baroreflex arc. It has been reported that total sleep deprivation can lead to reduced expression of nitric oxide synthase in the rat nodose ganglia (Chang et al., 2006). Therefore, chronic sleep deprivation may affect baroreceptor neurons in nodose ganglia. Although we have not ruled out the role of baroreceptor neurons in nodose ganglia in the reduction of BRS by sleep deprivation in the present study. However, we have found that neuronal activity in the NTS was altered in chronic sleep deprived rats compared with normal sleeping rats after inducing bradycardia using PE, while neuronal activity in the other nuclei related to the baroreflex circuit was not significantly altered. Therefore, we focused more on whether the NTS affected the BRS and its mechanism. In detail, the baroreflex mechanism involves peripheral baroreceptors sensing increases in arterial blood pressure and transmitting this information to the NTS. Furthermore, blood pressure is lowered by NTS neurons through activating cholinergic preganglionic parasympathetic neurons in the Amb and through activating GABAergic neurons in the CVLM, which in turn inhibit sympathoexcitatory adrenergic neurons in the RVLM. Which restores blood pressure homeostasis by decreasing heart rate and reducing vasomotor tone, respectively (Guyenet, 2006). In short, NTS, an integrative nucleus that communicate with main centers, is involved in autonomic control that participate in the increase sympathetic outflow and hypertension. The fact remains, however, that many brain regions play a role in regulating the baroreflex. In order to address this, other brain regions within the central nervous system should be studied more closely. Our further results showed that compared with control rats, the numbers of c-Fos positive neurons in the Amb, CVLM, and RVLM of rats with sleep deprivation were not changed significantly, while the number of c-Fos positive neurons in the NTS was increased significantly. In combination with NTS as the principal terminal site of the primary baroreceptor afferents. Therefore, we focus on the NTS in the brain regions associated with impaired BRS caused by sleep deprivation.

In the brain, the RAS plays a crucial role in the regulation of blood pressure and sympathetic tone (Marc and Llorens-Cortes, 2011). Ang-(1–7) is a key component of the renin-angiotensin system, which can counter-regulate several deleterious effects caused by Ang II. There is no denying that brain Ang-(1–7) has well-characterized actions in the regulation of arterial pressure and the BRS (Campagnole-Santos et al., 1989; Tsuda, 2013). In this study we have examined the concentrations of Ang II and Ang-(1–7) in the NTS tissues of control and sleep deprived rats. The results showed a significant increase in the concentration of Ang II in the NTS after sleep deprivation, while there was no significant difference in the concentration of Ang-(1–7). We consider that there is a problem in the balance between Ang II and Ang-(1–7), mainly involving enzymes involved in the metabolism of Ang II and Ang-(1–7), such as ACE, ACE2, and dipeptidyl peptidase 3. In this study, we have not detected the expression and activity of enzymes involved in Ang II and Ang-(1–7) metabolism, which requires our more attention in future study. Another intriguing finding in the present study is that activation of the AT1R by Ang II is upstream to the induced BRS impairment under sleep disorder. In this study, although we have not tested the effects of microinjection of Ang II in NTS on BRS in control rats. However, previous studies have shown that microinjections of Ang II in NTS could blunt the baroreflex in control rats (Casto and Phillips, 1986; Luoh and Chan, 2001). These results suggest that the brain Ang II plays an important role in sleep deprivation-depressed baroreflex. To further detect whether AT1R with increased expression in the NTS mediates BRS damage induced by sleep deprivation, we observed the effect of blocking AT1R on BRS impairment induced by sleep deprivation by microinjection *in vivo*. We found that selective blockade of AT1R in the NTS by losartan significantly reversed the inhibition of BRS caused by sleep deprivation. Based on our findings, AT1R in the NTS plays a critical role in long-term sleep deprivation-induced BRS impairment. However, a shortcoming of this study is that no in-depth experiments have been conducted to clarify the molecular mechanism involved in BRS impairment after activating AT1R under sleep deprivation. Interestingly, it has also been shown that Ang II activates AT1R in non-neuronal cells and brain nerve cells, thereby inducing the expression of c-Fos gene (Chan et al., 1998). Furthermore, another study found that Ang II inhibited baroreflex through the expression of c-Fos in the NTS of rats (Chan et al., 2002). These findings suggest that c-Fos protein expression by AT1R may be responsible for Ang II inhibiting BRS. Astonishingly, our study also found a significant increase in the number of c-Fos positive neurons in the NTS in sleep-deprived rats. These results of our study, as well as those of other groups, will help us speculate that AT1R activated in NTS after sleep deprivation damages BRS by regulating c-Fos expression.

The most important finding of this study is that the neurons subserving the baroreflex in the NTS appear to be chronically activated under chronic sleep deprivation. This supports the concept that the baroreflex has a sustained suppressive influence on cardiovascular effects induced by sleep deprivation. However, another limitation of the present study deserves consideration. It was unclear in this study which type of neurons in the NTS are responsible for sleep deprivation-induced BRS damage, so AT1R was found to play a role in BRS damage in response to sleep deprivation. Our study found that the number of c-Fos positive neurons activated by PE in the NTS of rats increased significantly after chronic sleep deprivation. In the NTS, baroreflex-sensitive neurons were primarily glutaminergic excitatory neurons. Some studies also proved that some of the baroreflex-sensitive neurons labeled by PE were inhibitory GABAergic neurons (about 20%) (Yao et al., 2022). However, we found no significant changes in other nuclear groups in the baroreflex-regulating regions, so we believe that the neurons with increased PE activation in the NTS after sleep deprivation may not be all glutaminergic neurons.

In summary, combined with the effects of microinjection of losartan into the NTS on impaired BRS induced by chronic sleep deprivation in this study, we have revealed the role and significance of abnormal RAS in the NTS for regulating the BRS under chronic sleep loss. These data facilitate understanding a new insight into the mechanism of BRS impairment induced by sleep deprivation. Furthermore, these findings suggest the inhibition of the elevated AT1R expression in the NTS exerted cardiovascular protective effects, which might be a novel therapy approach for preventing and treating the cardiovascular diseases and their risk factors caused by chronic sleep disorder.

## Data availability statement

The original contributions presented in the study are included in the article/Supplementary material, further inquiries can be directed to the corresponding authors.

## Ethics statement

The animal study was approved by the Animal Use and Care Committee of the Naval Medical University. The study was conducted in accordance with the local legislation and institutional requirements.

## Author contributions

L-fL: Data curation, Formal analysis, Investigation, Methodology, Writing – original draft. Y-wW: Data curation, Investigation, Validation, Writing – review & editing. J-cS: Conceptualization, Project administration, Supervision, Validation, Writing – review & editing. Y-kW: Formal analysis, Validation, Visualization, Writing – review & editing. XT: Conceptualization, Funding acquisition, Writing – original draft. W-zW: Funding acquisition, Investigation, Validation, Visualization, Writing – review & editing.
