# Supplementary figures and images for: Sleep deprivation reduces the baroreflex sensitivity through elevated angiotensin (Ang) II subtype 1 receptor expression in the nucleus tractus solitarii
Source: Front Neurosci. 2024 Apr 29;18:1401530. doi: 10.3389/fnins.2024.1401530 (PMC11089155; doi:10.3389/fnins.2024.1401530)

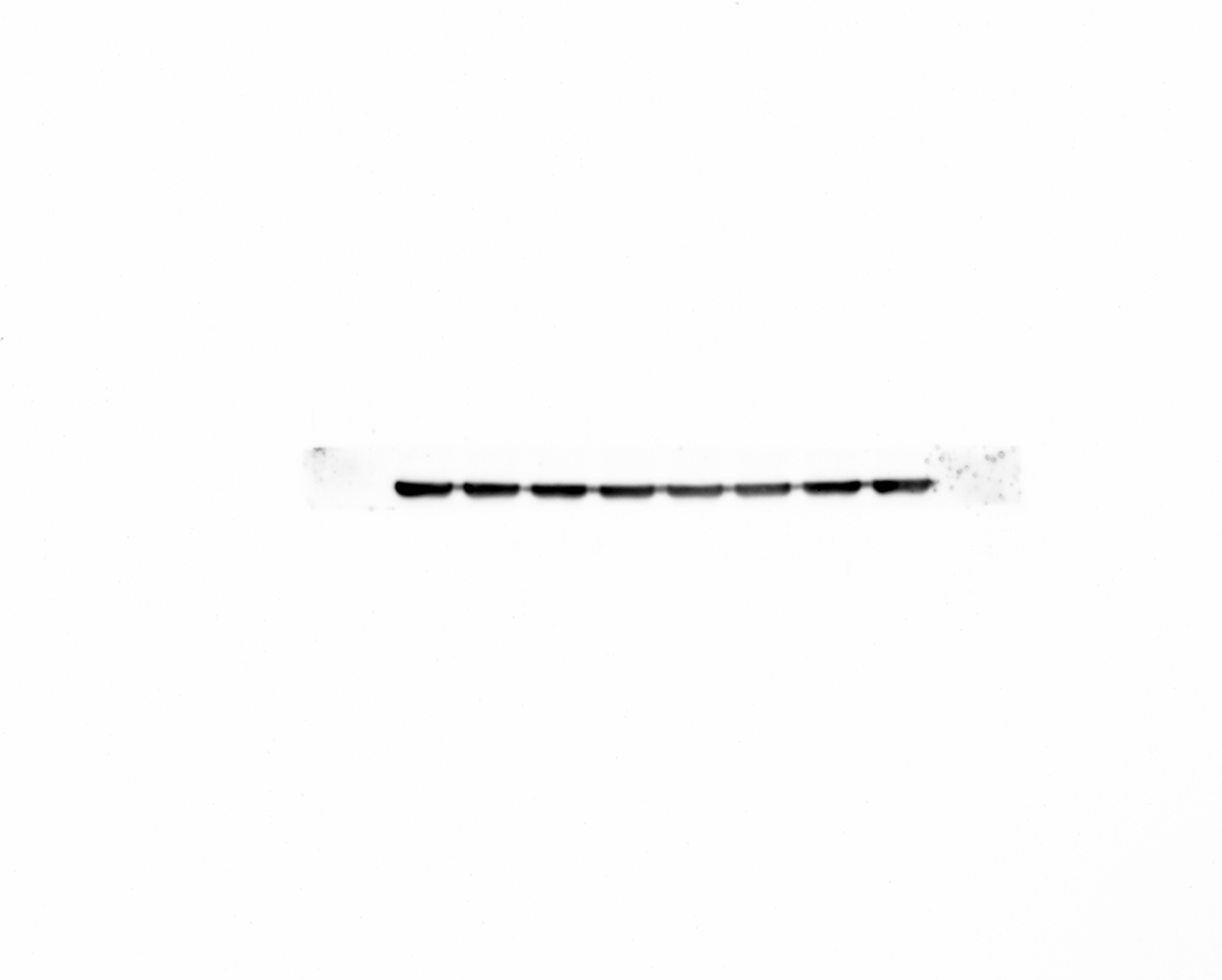

Supplement: Supplementary file 2 [file Data_Sheet_1.ZIP › wb supplement/1-2-a┬-Tubulin.tif]

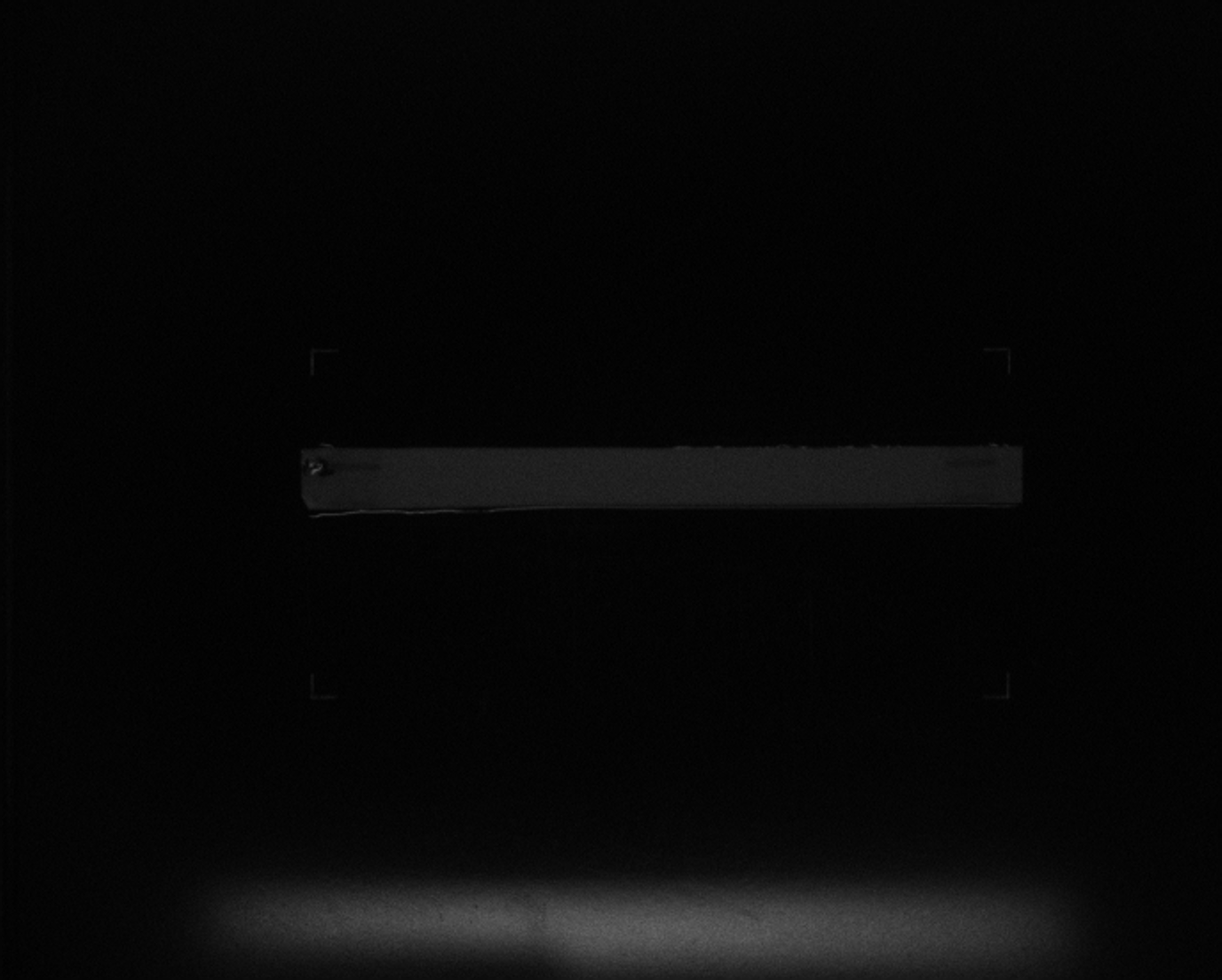

Supplement: Supplementary file 2 [file Data_Sheet_1.ZIP › wb supplement/1-2a┬-Tubulin-Marker.tif]

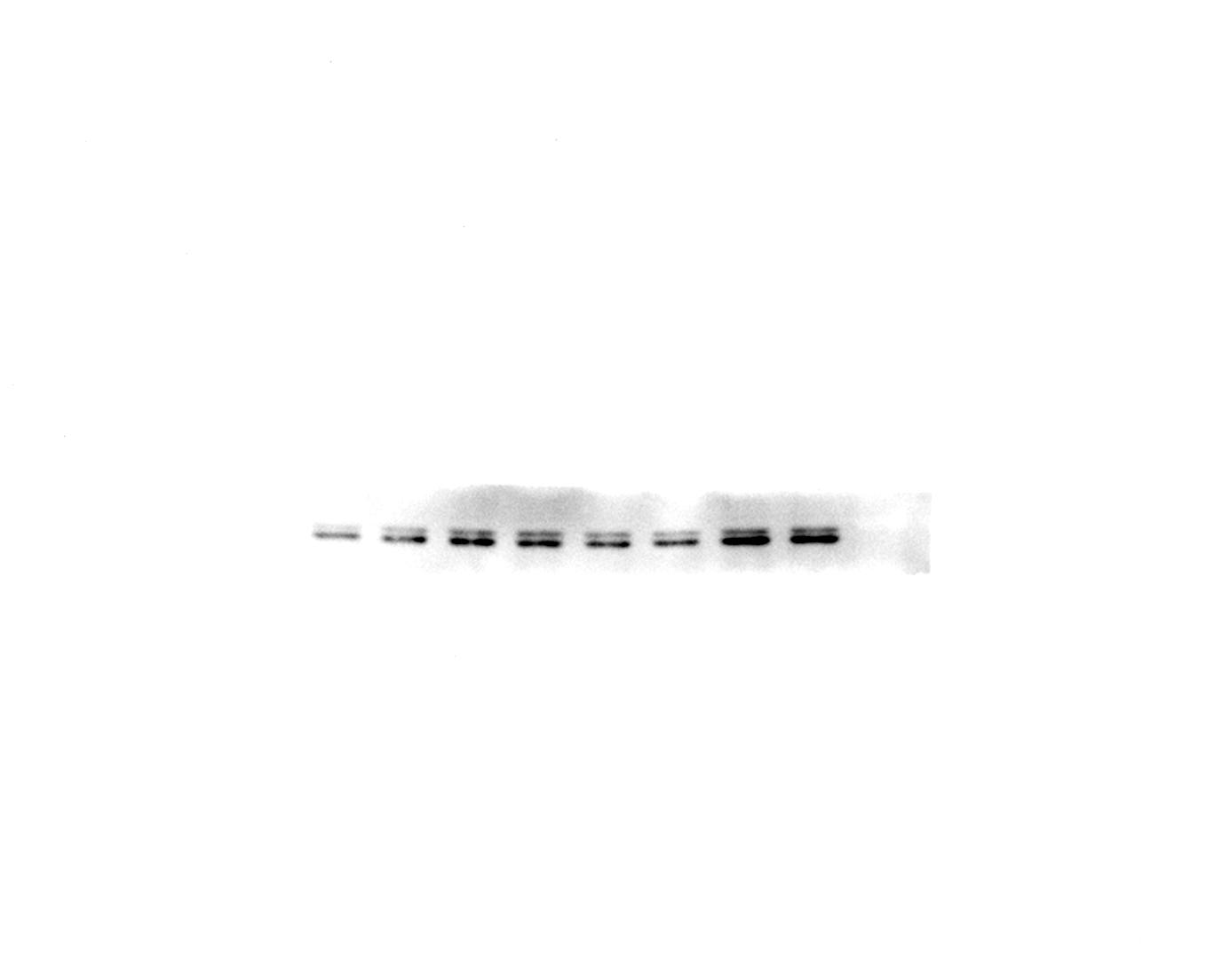

Supplement: Supplementary file 2 [file Data_Sheet_1.ZIP › wb supplement/1-3-AT1R.tif]

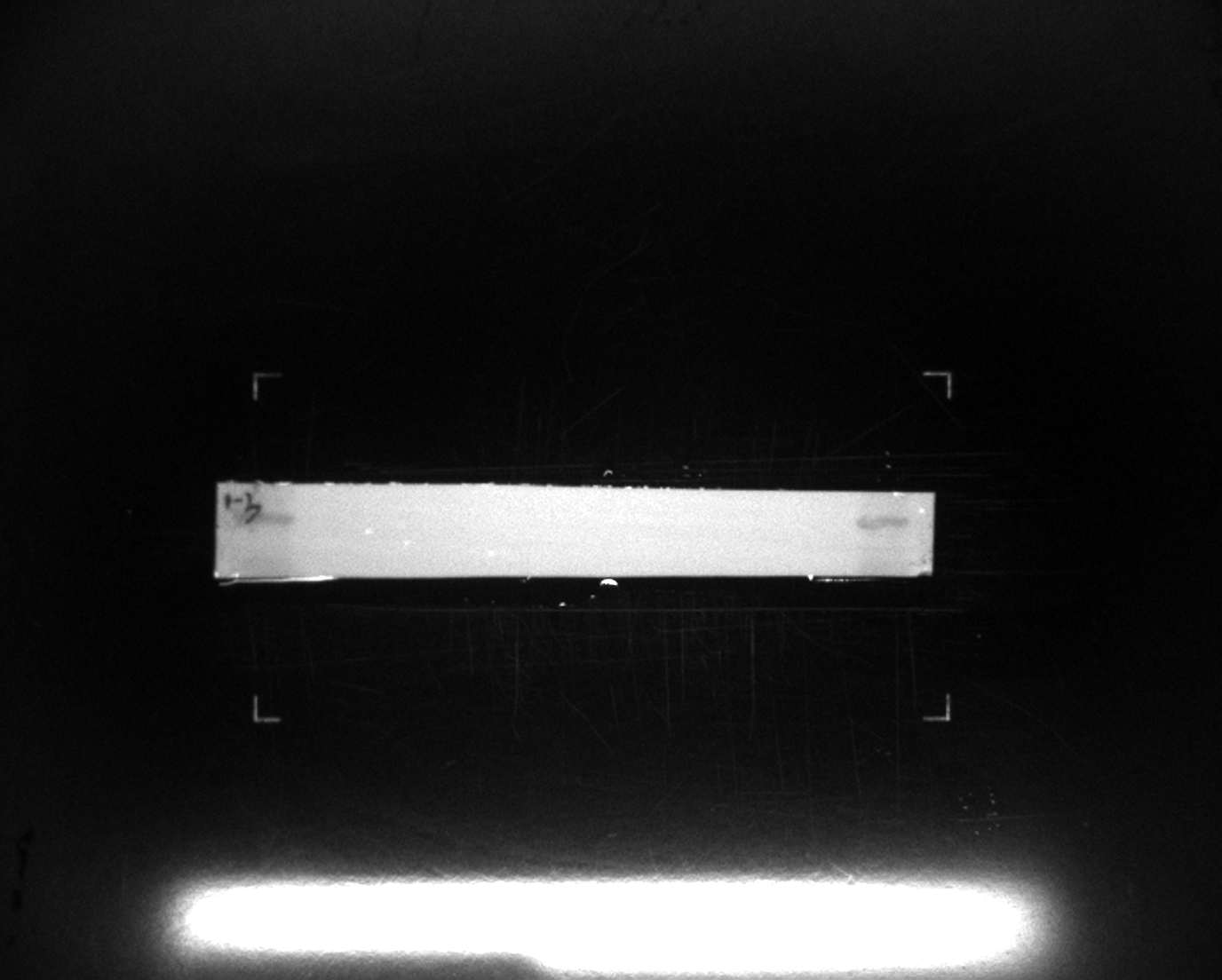

Supplement: Supplementary file 2 [file Data_Sheet_1.ZIP › wb supplement/1-3-AT1R-Marker.tif]

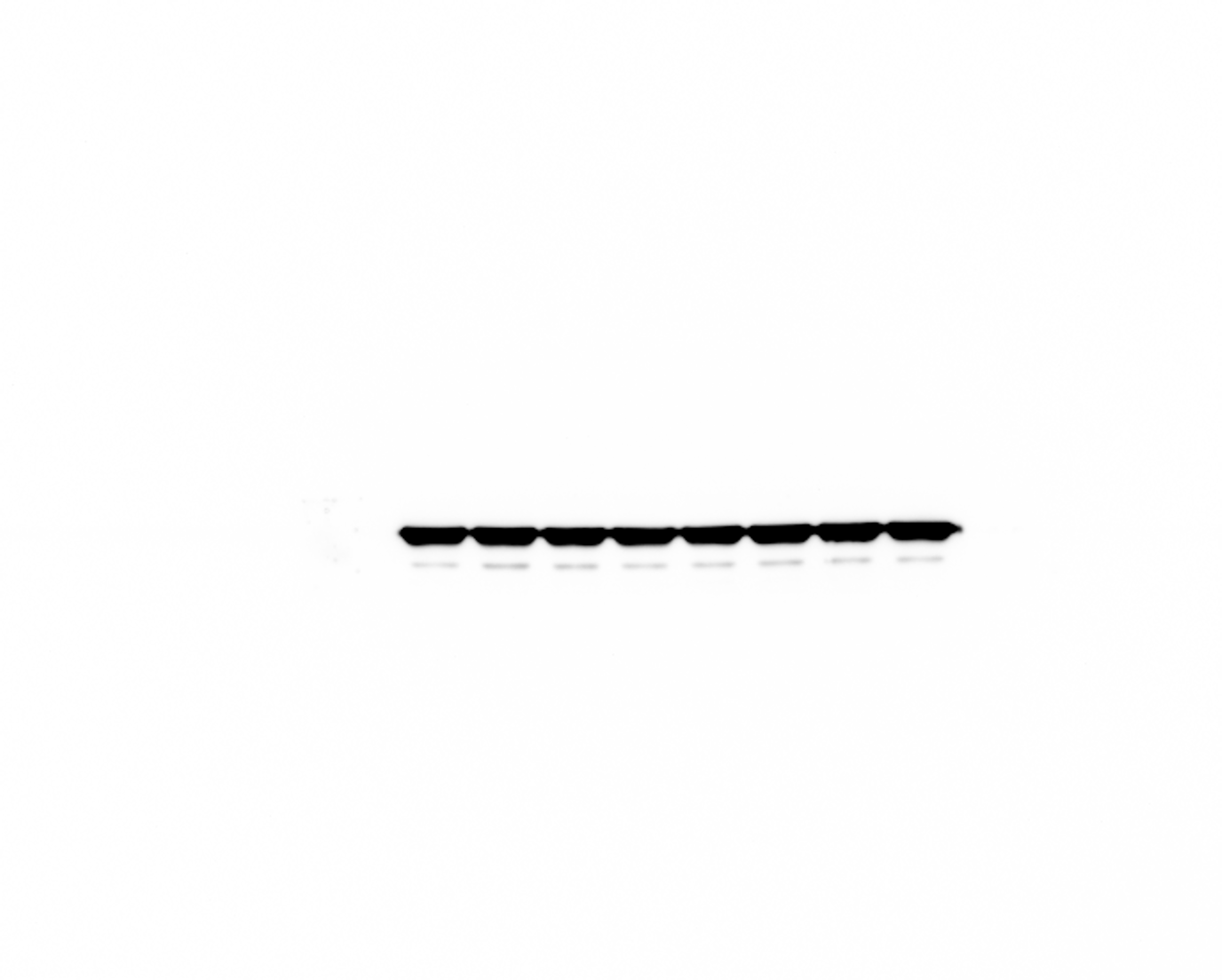

Supplement: Supplementary file 2 [file Data_Sheet_1.ZIP › wb supplement/2-2-a┬-Tubulin.tif]

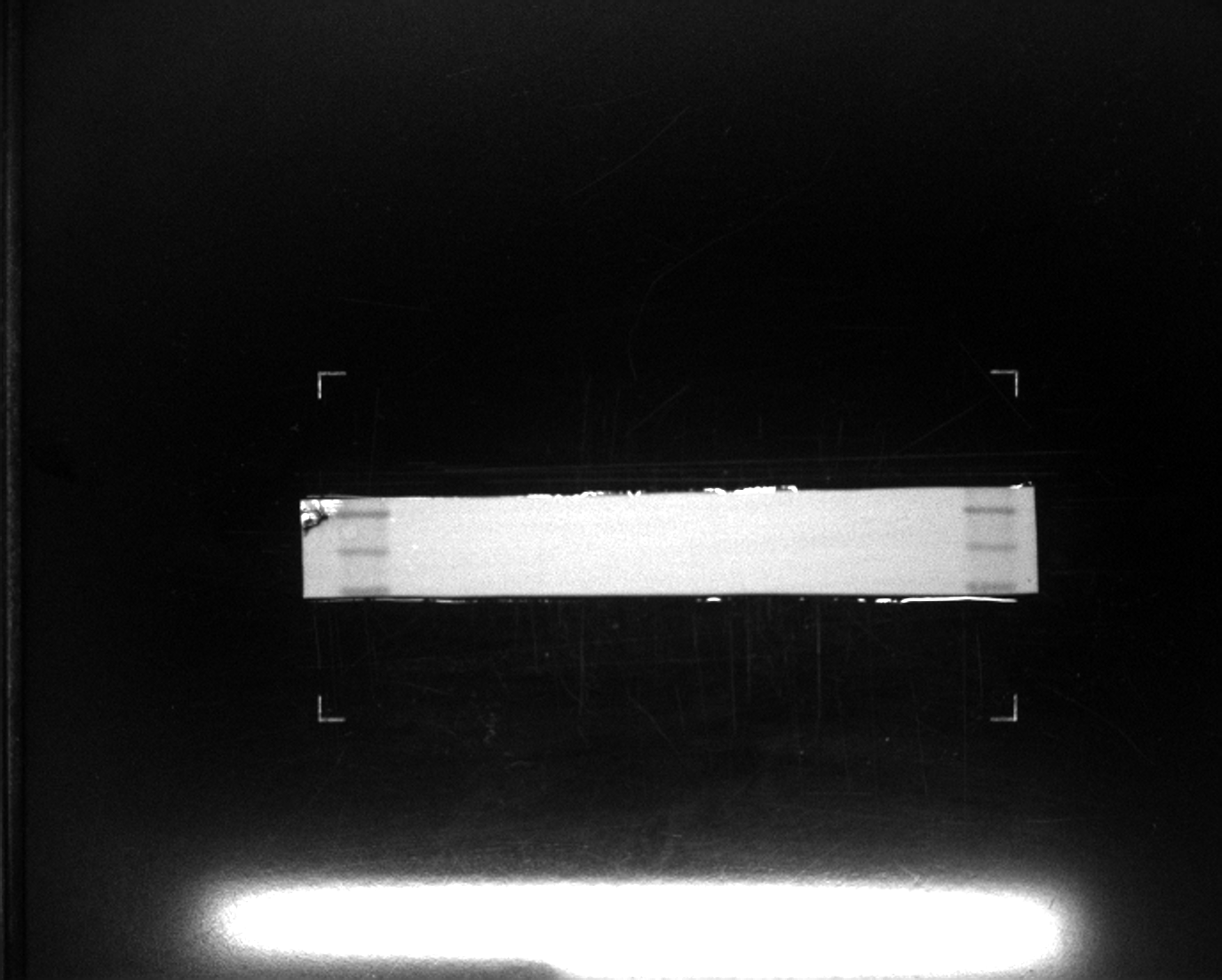

Supplement: Supplementary file 2 [file Data_Sheet_1.ZIP › wb supplement/2-2-a┬-Tubulin-Marker.tif]

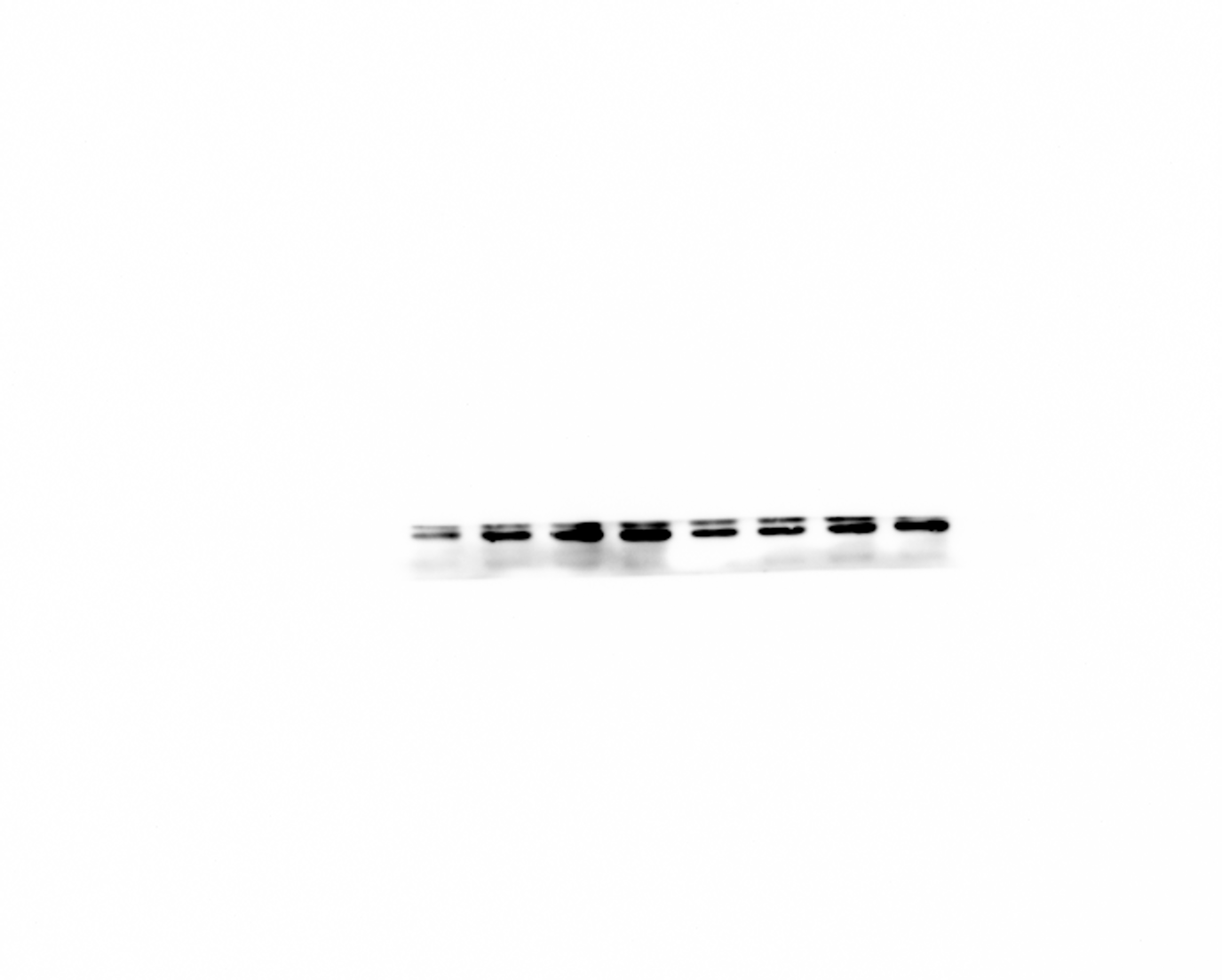

Supplement: Supplementary file 2 [file Data_Sheet_1.ZIP › wb supplement/2-3-AT1R.tif]

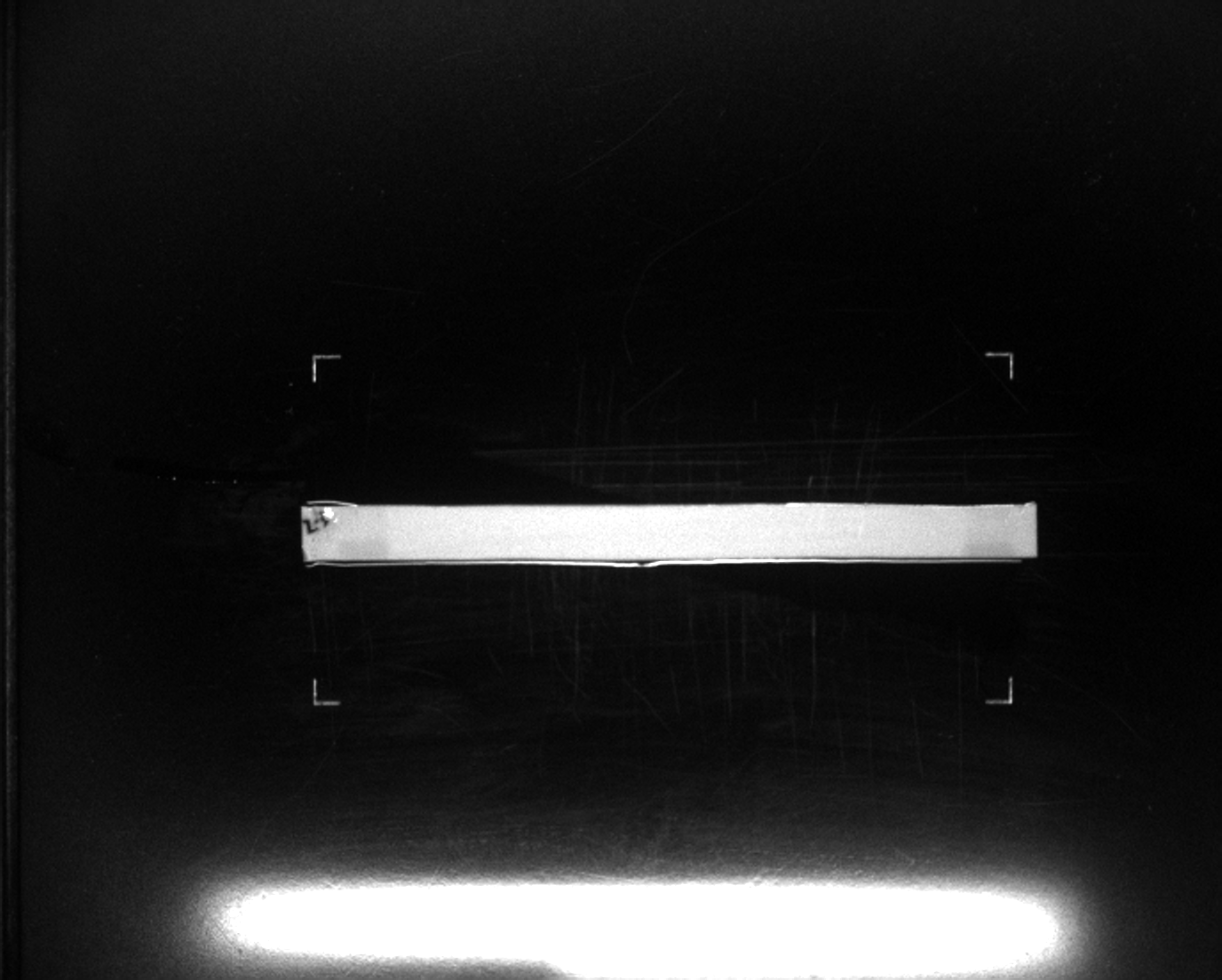

Supplement: Supplementary file 2 [file Data_Sheet_1.ZIP › wb supplement/2-3AT1R-Marker.tif]

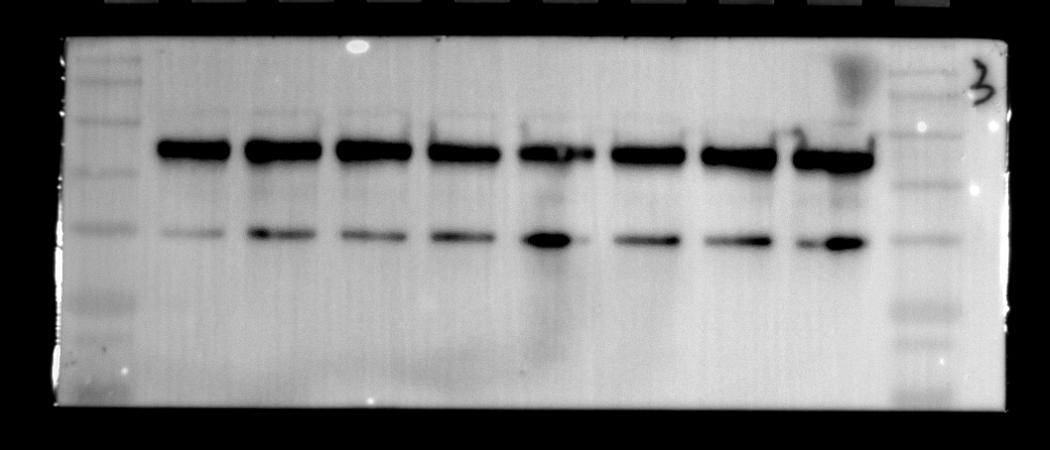


63KDa

KDa

48KDa

KDa

35KDa

CSD

Ctrl

Supplement: Supplementary file 3 [file Data_Sheet_2.ZIP › AT1R WB(1).docx]
